# A Request

**Published:** 1916-09

**Authors:** 


### A Request.

Doctor, if you have finished with your August, 1916, copy of THE TEXAS MEDICAL JOURNAL, will you please send it back to the editor? A two-cent stamp will bring it and the courtesy will be greatly appreciated. The issue was so popular the demand exceeded the supply. Do it today.

